# A Survey of Cancer Risk Behaviors, Beliefs, and Social Drivers of Health in New Hampshire and Vermont

**DOI:** 10.1158/2767-9764.CRC-23-0267

**Published:** 2023-08-29

**Authors:** Thomas A. Skipper, Julie E. Weiss, Heather A. Carlos, Christine M. Gunn, Rian M. Hasson, Janet L. Peacock, Jenna E. Schiffelbein, Anna N.A. Tosteson, Frederick Lansigan, Judy R. Rees

**Affiliations:** 1 Dartmouth Cancer Center, Lebanon, New Hampshire.; 2 Department of Molecular and Systems Biology, Geisel School of Medicine at Dartmouth, Hanover, New Hampshire.; 3 The Dartmouth Institute for Health Policy and Clinical Practice, Geisel School of Medicine at Dartmouth, Hanover, New Hampshire.; 4 Department of Surgery, Section of Thoracic Surgery, Dartmouth-Hitchcock Medical Center, Lebanon, New Hampshire.; 5 Department of Epidemiology, Geisel School of Medicine at Dartmouth, Hanover, New Hampshire.; 6 Department of Hematology, Dartmouth-Hitchcock Medical Center, Lebanon, New Hampshire.

## Abstract

Compared with urban areas, rural areas have higher cancer mortality and have experienced substantially smaller declines in cancer incidence in recent years. In a New Hampshire (NH) and Vermont (VT) survey, we explored the roles of rurality and educational attainment on cancer risk behaviors, beliefs, and other social drivers of health. In February–March 2022, two survey panels in NH and VT were sent an online questionnaire. Responses were analyzed by rurality and educational attainment. Respondents (*N* = 1,717, 22%) mostly lived in rural areas (55%); 45% of rural and 25% of urban residents had high school education or less and this difference was statistically significant. After adjustment for rurality, lower educational attainment was associated with smoking, difficulty paying for basic necessities, greater financial difficulty during the COVID-19 pandemic, struggling to pay for gas (*P* < 0.01), fatalistic attitudes toward cancer prevention, and susceptibility to information overload about cancer prevention. Among the 33% of respondents who delayed getting medical care in the past year, this was more often due to lack of transportation in those with lower educational attainment (21% vs. 3%, *P* = 0.02 adjusted for rurality) and more often due to concerns about catching COVID-19 among urban than rural residents (52% vs. 21%; *P* < 0.001 adjusted for education). In conclusion, in NH/VT, smoking, financial hardship, and beliefs about cancer prevention are independently associated with lower educational attainment but not rural residence. These findings have implications for the design of interventions to address cancer risk in rural areas.

**Significance:**

In NH and VT, the finding that some associations between cancer risk factors and rural residence are more closely tied to educational attainment than rurality suggest that the design of interventions to address cancer risk should take educational attainment into account.

## Introduction

Cancer is the second leading cause of death in the United States, with 1.9 million new cases and 609,360 deaths expected in 2022 (1). The burden is increasingly unequal; despite recent decreases in cancer mortality nationally, rural populations and those with lower educational attainment have experienced little change (2–6). Almost one in five Americans live in rural areas and may be affected by this disparate share of the cancer burden (7). The causes are complex and have been shown to be related to differences in cancer risk behaviors, beliefs, and social drivers of health (8).

Rural areas generally have higher prevalence of cigarette smoking and obesity compared with large metropolitan areas (9–11). Disadvantageous beliefs, such as fatalism, are also more common among rural populations and those with lower educational attainment (12–13). Fatalism is characterized by feelings of powerlessness to prevent or survive cancer and believing that external forces are in control (13). Individuals with these beliefs are less likely to engage in preventive behaviors like cancer screening, healthy diet, exercise, and smoking cessation (14–17). Rural populations have poorer access to medical care, local providers, and transportation, and are more likely to be older, of lower educational attainment, of lower socioeconomic status, and underinsured (18–21). Many of these trends in cancer risk behaviors, beliefs, and social drivers of health are also seen in those with lower educational attainment (6). Although other factors such as socioeconomic status and social environment have been compared in parallel with education, there has been less emphasis on evaluating the independent and joint effects of education and rurality on cancer risk factors (22–23). One recent article that found an interaction between rurality and education investigated differences in survival in patients with ovarian cancer and not general cancer risk factors (24).

The Dartmouth Cancer Center's (DCC) Community Outreach and Engagement (COE) team investigated rural residence and educational attainment and their associations with cancer risk factors in the DCC catchment area states of New Hampshire (NH) and Vermont (VT). NH ranks 8th in the nation for cancer incidence overall and ranks in the top three states for seven cancer types (bladder,^,^ brain, breast, esophageal, and childhood cancer, as well as Hodgkin lymphoma and melanoma); VT ranks 17th for incidence and is in the top three states for three cancer types (melanoma, and cancers of the testis and uterus; refs. 25–27). Although rural counties have been shown to have lower incidence and higher mortality than urban counties (28), NH has significantly higher incidence but not mortality than the rest of the Unites States, and VT has significantly higher mortality but not lower incidence (25–27). The many factors that appear to drive the disproportionate cancer burden associated with rural residence have been shown to vary by region (3–6, 14–17). In our region, most of the health professional shortage areas and medically underserved areas in NH (78% and 60%, respectively) and VT (93% and 90%) are designated as rural by the Health Resources and Services Administration (29). This article is the first to explore the independent and synergistic roles of education and rurality in the context of cancer disparities in NH and VT—small, racially homogeneous states that are often overlooked in analyses of disparities. We hypothesized that there are differences in cancer risk behaviors, beliefs, and social drivers of health by rural (versus urban) residence and by educational attainment.

## Materials and Methods

The DCC is the only Comprehensive Cancer Center designated by the NCI in NH and VT, its catchment area. We conducted an online survey of the DCC catchment population using a web-based survey of Granite State Panel and Green Mountain State Panel members recruited from randomly-selected landline and cell phone numbers across NH and VT. The Granite State Panel and Green Mountain State Panel are part of an effort by the University of New Hampshire Survey Center to investigate new ways of gathering and understanding the opinion of NH and VT residents (30). Respondents to UNH Survey Center surveys were asked whether they wished to participate in further research and asked to provide an email address. Those who agreed and provided an email address were added to the panel. Panel members were also recruited by texting a random sample of cellular telephones in the state and inviting the recipient to take a short survey. At the conclusion of the survey, recipients were asked whether they would like to join the panel and provide an email address. Respondents under the age of 18, non-state residents, and seasonal residents who are not registered to vote in that state were excluded from this survey and did not receive an invitation to join the panel. For each completed survey, panel members are entered into quarterly drawings to earn rewards, such as gift certificates from statewide and internet companies.

The survey (Supplementary Table S1) was offered to these two panels between February 25, 2022 and March 8, 2022, with the goal of attaining the *a priori* target of 1,700 completed surveys (1,000 in NH and 700 in VT). Two email reminders were sent to nonresponders 1 and 3 days after the invitation and the survey was closed on the morning of March 8th. We elicited sociodemographic information and responses to questions about cancer risk behaviors, cancer attitudes and beliefs, and social drivers of health including health care access and financial, food, and transportation security.

The study was conducted according to recognized ethical guidelines; use of human subjects for the Granite State and Green Mountain State Panels was approved by the Institutional Review Board for the University of New Hampshire (Durham, NH), and this study was approved by the Dartmouth College Committee for the Protection of Human Subjects following the ethical principles identified in the Belmont report. Participants provided written consent electronically within the online survey.

### Statistical Analysis

Each survey response from the NH and VT population was weighted by the respondent's age, gender, education, and region of the state using targets from the most recent U.S. Census Bureau's American Community Survey conducted by the U.S. Census Bureau, as well as political party registration levels in NH and 2020 election results. NH regions used for weighting are the Central/Lakes Region (Belknap, Merrimack Counties); Hillsborough County; Northern NH (Carroll, Coos, Grafton Counties); Seacoast (Rockingham, Strafford Counties); and Western NH (Cheshire, Sullivan Counties). VT regions are Central VT (Addison, Orange, Washington Counties), Chittenden County; Northern Counties (Caledonia, Essex, Franklin, Grand Isle, Lamoille, Orleans Counties); Southern VT (Bennington, Rutland, Windham, Windsor Counties). Like all surveys, this survey is subject to sampling error due to the fact that all residents in the area were not interviewed. For those questions asked of 500 or so respondents, the error is ±4.4%. For those questions where fewer than 500 persons responded, the sampling error can be calculated as ±1.96√[*P*(1 − *P*)/*N*] where *P* is the percentage of responses in the answer category being evaluated and *N* is the total number of persons answering the particular question. The margin of sampling error for our survey is ±2.6%. The design effect for the survey is 2.9%. Because of rounding, percentages may not sum to 100%. The number of respondents in each demographic below may not equal the number reported in cross-tabulation tables as some respondents choose not to answer some questions.

We analyzed results based on residence (rural vs. urban) and educational attainment (lower vs. higher). Rural and urban areas were defined using Rural and Urban Commuting Areas (RUCA); primary RUCA codes 1–3 (Metropolitan) were classified as urban while 4–10 were considered rural (31). Educational attainment was classified as “lower” for those with high school graduation or less and “higher” for those with any education beyond high school. Statistical comparisons examining differences by rurality and by educational attainment were made based on weighted frequencies and means using Rao-Scott *χ*^2^ tests of independence and *t* tests, respectively. Domain analysis was used to account for variability of the whole sample to estimate the variance of subgroups. Totals (*N*) and percentages (%) may not equal 100% due to rounding. Multivariable models assessed the independent associations on each cancer risk factor of educational attainment (high school or less vs. more than high school) and rural (vs. urban) residence. An interaction term (education × rurality) was included in each model and dropped when not statistically significant. Unadjusted and adjusted *P* values are presented for each association tested. Analyses used SAS version 9.4 (SAS Institute Inc.; ref. 32).

### Data Availability Statement

The datasets generated and analyzed from the population survey are not publicly available at the time of publication, as they are still being analyzed for other articles; however, they may be available in the future from the corresponding author on reasonable request.

## Results

**FIGURE 1:**
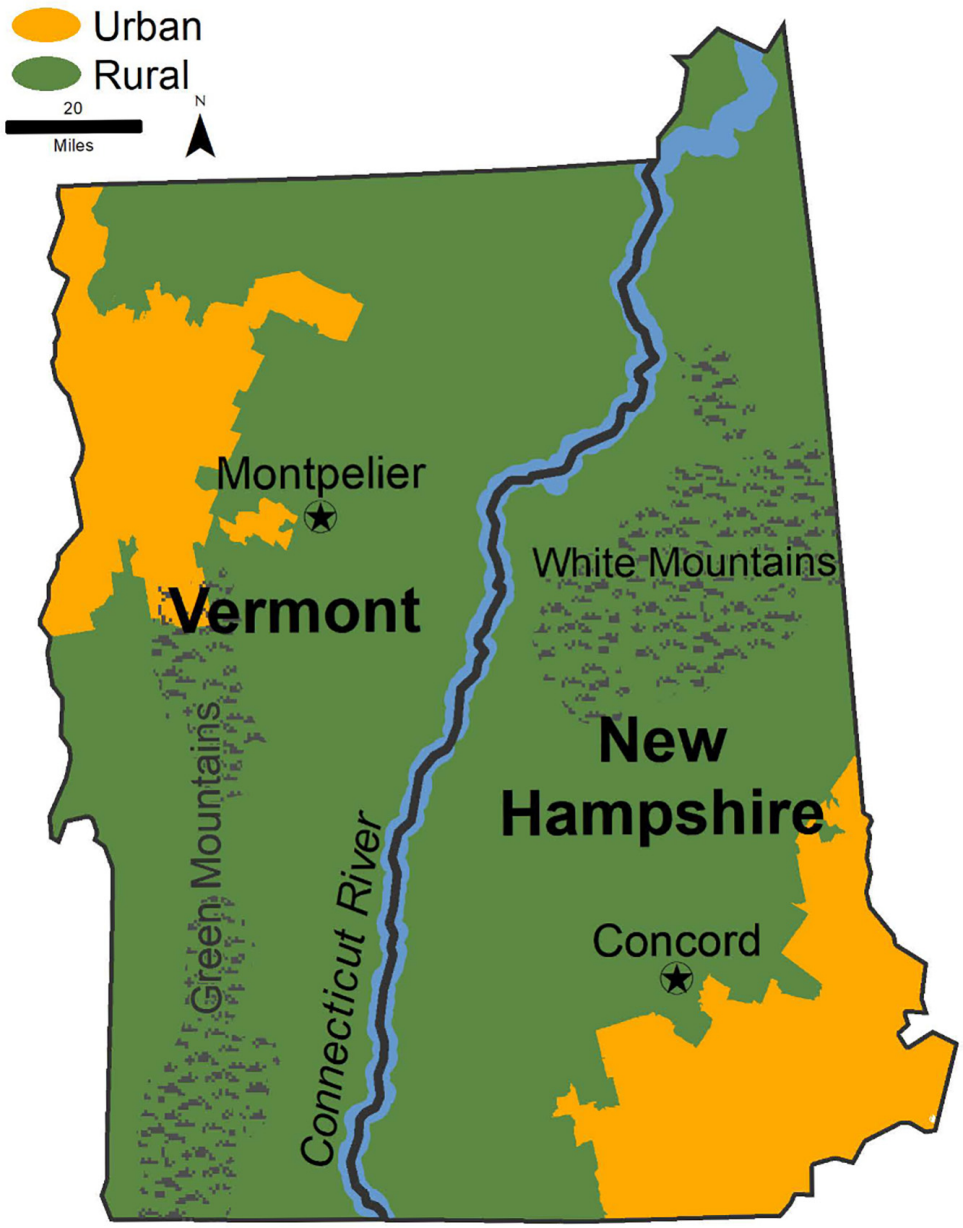
NH and VT maps showing rural and urban regions. Rural regions are defined using RUCA codes 4–10 and urban using RUCA 1–3.

The 1,717 survey responses represented a response rate of 27%. When comparing responders and nonresponders, some differences were seen by gender, political party affiliation, education, and age (Supplementary Table S2), but these factors were taken into account by the weighting process. Most participants identified as white (96%), consistent with the overall demographics of the two state populations. Fifty percent were women; 48% were men; and 2% identified as transgender, gender expansive, or preferred not to say (Table 1). More than half (55%, *n* = 939) lived in rural areas, and 36% (*n* = 618) had a high school education or less (≤HS). The distributions of rural and urban residence differed in the two states, with a greater proportion of rural residence in VT (Fig. 1). Lower educational attainment was significantly more prevalent in rural than urban areas (45% vs. 25%, *P* = 0.001), and lower salaries were more common among rural participants (vs. urban, *P* = 0.02) and those with lower educational attainment (vs. higher, *P* = 0.04). Rural participants were significantly more likely than urban participants to be insured through Medicaid (20% vs. 9%). Nine percent reported a history of cancer.

**TABLE 1:** Survey participant characteristics by rurality (rural vs. urban) and educational attainment (high school or less vs. more than high school). 1,717 NH and VT residents were surveyed by phone in February–March 2022

		Rural residence				Educational attainment			
		Total *N* = 1,700	Rural *N* = 939	Urban *N* = 761		Total *N* = 1,709	≤HS *N* = 618	>HS *N* = 1,091	
Participant characteristics		*N* (%)	%	%	*P*	*N* (%)	%	%	*P*
State	Both	1,700 (100)	55%	45%		1,709 (100)	36%	64%	
	New Hampshire	1,002 (59)	42%	79%	<0.0001	1,008 (59)	59%	59%	0.94
	Vermont	698 (41)	58%	21%		701 (41)	41%	41%	
Rurality	Rural	939 (55)	100%		—	935 (55)	69%	47%	0.001
	Urban	761 (45)		100%		757 (45)	31%	53%	
Age	18 to 34	462 (27)	27%	28%	0.81	462 (27)	27%	28%	0.77
	35 to 49	330 (20)	20%	19%		335 (20)	17%	22%	
	50 to 64	521 (31)	29%	34%		525 (31)	35%	28%	
	65 and older	373 (22)	24%	20%		374 (22)	21%	22%	
Race	White	1,586 (96)	96%	95%	0.39	1,600 (96)	98%	95%	0.12
	Non-White	68 (4)	4%	5%		618 (37)	2%	5%	
Ethnicity	Non-Hispanic	1,672 (99)	99%	98%	0.27	1,686 (99)	100%	98%	
	Hispanic	23 (1)	1%	2%		23 (1)	2%	0%	
Gender	Women	844 (50)	48%	52%	0.24	854 (50)	46%	53%	0.66
	Men	817 (48)	49%	47%		821 (48)	52%	46%	
	Other	25 (2)	2%	0%		25 (1)	2%	1%	
Education	High school or less	613 (36)	45%	25%	0.001	618 (36)	100%		—
	Technical school/some college	500 (30)	26%	33%		508 (30)		47%	
	College	353 (21)	17%	26%		354 (21)		32%	
	Post graduate	226 (13)	12%	16%		228 (13)		21%	
Marital status	Married	995 (59)	56%	62%	0.59	1,007 (59)	58%	60%	0.95
	Divorced/separated	229 (14)	15%	12%		230 (14)	14%	13%	
	Never married	468 (28)	29%	26%		468 (27)	28%	27%	
Household size	1–2 people in household	1,093 (65)	68%	61%	0.25	1,099 (65)	71%	61%	0.20
	3+ people in household	598 (35)	32%	39%		601 (35)	29%	39%	
Home	Owner	1,208 (73)	69%	77%	0.17	1,214 (73)	61%	79%	0.01
	Renter/Other	458 (27)	31%	23%		460 (27)	39%	21%	
Household income	<$45,000	327 (23)	26%	20%	0.02	327 (23)	25%	22%	0.04
	$45,000–$74,999	331 (24)	29%	16%		336 (24)	27%	22%	
	$75,000–$99,999	257 (18)	15%	22%		258 (18)	18%	18%	
	$100,000–$149,999	374 (27)	24%	31%		378 (27)	29%	25%	
	$150,000 or more	111 (8)	5%	12%		112 (8)	0%	13%	
Insurance	Employer	985 (58)	49%	69%	<0.001	994 (58)	51%	62%	0.07
	Exchange or Other	155 (9)	11%	7%		155 (9)	11%	8%	
	Medicaid	258 (15)	20%	9%		257 (15)	23%	10%	
	Medicare	271 (16)	18%	13%		273 (16)	13%	17%	
	Military	14 (1)	1%	1%		14 (1)	1%	1%	
	None	16 (1)	1%	1%		16 (1)	0%	1%	
Had cancer	No	1,539 (91)	92%	90%	0.64	1,542 (91)	91%	90%	0.84
	Yes	152 (9)	8%	10%		157 (9)	9%	10%	

NOTE: Missing (*N*): **Rurality:** Rurality (17), Age (13), Race (46), Gender (14), Education (7), Marital Status (8), Number of people in household (26), Household income (301) Help with written material (11), Ever had cancer (9). **Education:** Education (8), Rurality (16), Age (13), Race (41), Gender (10), Marital Status (4), Number of people in household (18), Household Income (299), Insurance (1), Help with written material (11), Ever had cancer (9). Other (Transgender, Gender expansive, prefer not to say).

Abbreviations: HS, high school.

For the cancer risk factors we assessed, we more often found significant independent associations with educational attainment. When we saw univariate associations between risk factors and rurality, these were not generally significant after adjustment for educational attainment. Education by rurality interaction terms were not significant in any model and were removed.

**FIGURE 2:**
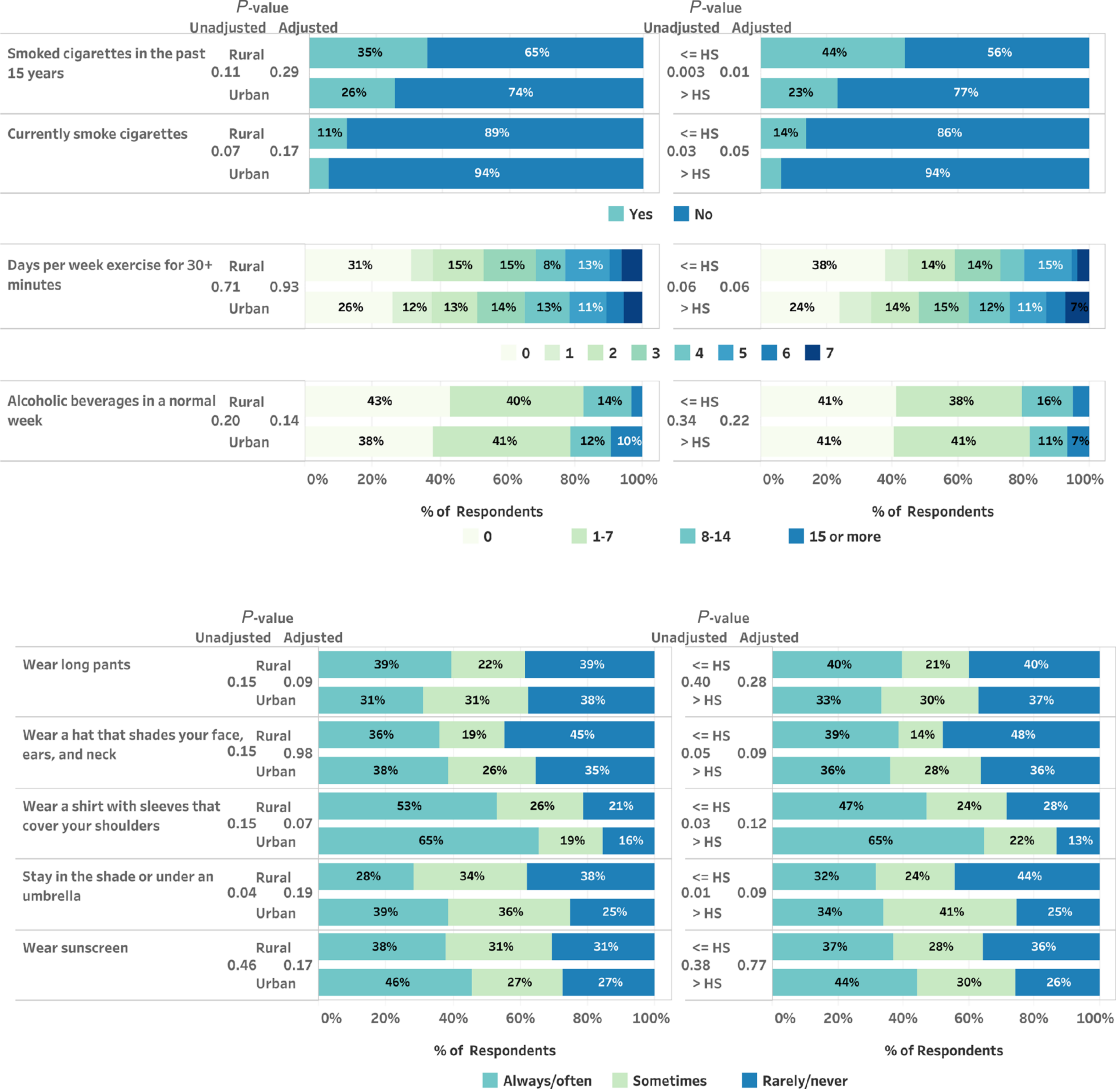
Cancer risk factors by rurality (rural vs. urban) and educational attainment [≤ high school (HS) vs. > HS] among 1,717 NH and VT residents surveyed in February–March 2022.

Overall, 31% had smoked within the past 15 years, and 9% were current smokers (Fig. 2; Supplementary Table S3). Compared with those with more than high school education, participants with lower educational attainment were more likely to be current smokers (14% vs. 6%, unadjusted *P* = 0.03, adjusted for rurality *P* = 0.05) or to have smoked in the past 15 years (44% vs. 23%, unadjusted *P* = 0.003, adjusted for rurality *P* = 0.01). Rural residence and smoking status were not significantly associated. No significant differences were seen for alcohol use or exercise by rurality or educational attainment. Although the use of sunscreen was similar by rurality and education, those with higher (vs. lower) educational attainment were more likely to protect themselves by wearing a long-sleeved shirt and more likely to stay in the shade (Fig. 2).

**FIGURE 3:**
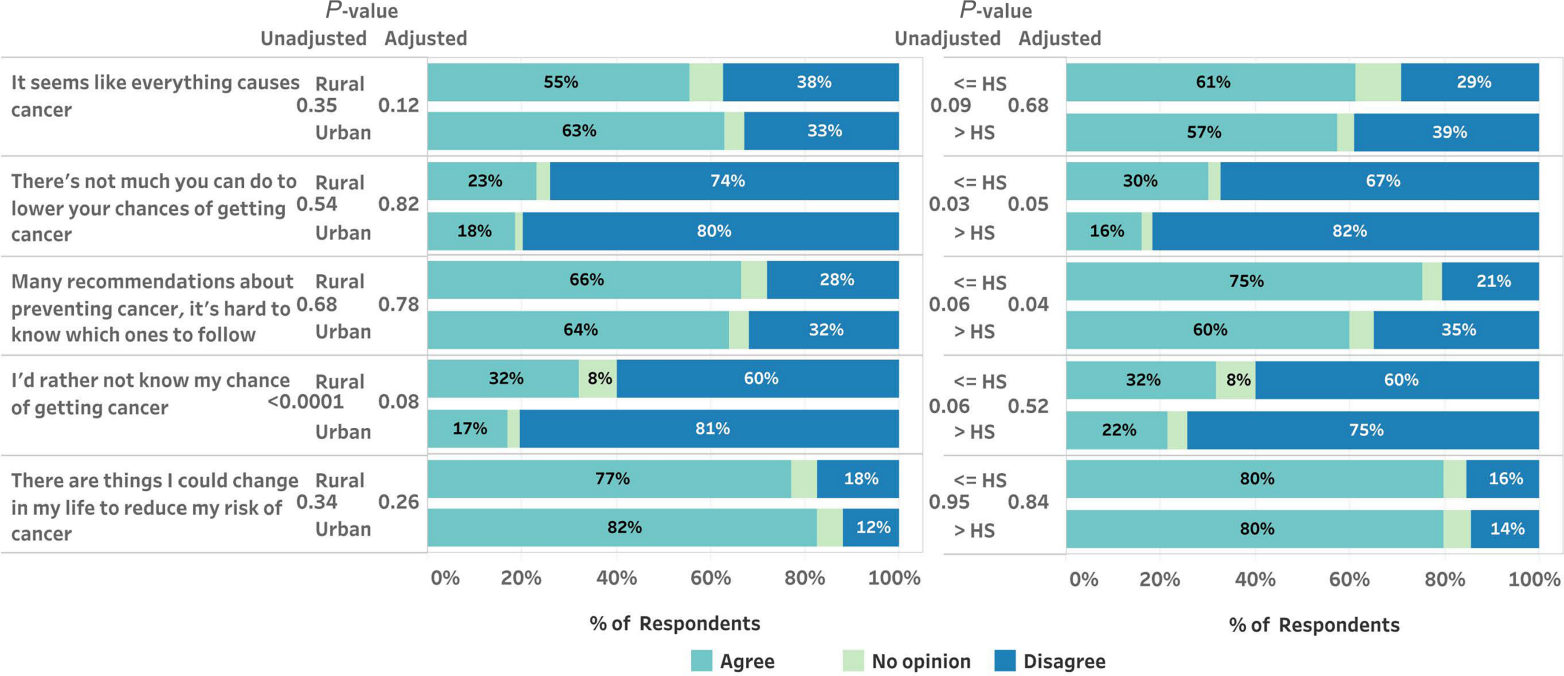
Cancer beliefs by rurality (rural vs. urban) and educational attainment [≤ high school (HS) vs. > HS] among 1,717 NH and VT residents surveyed in February–March 2022.

When asked their opinions about cancer, 30% of those with lower educational attainment agreed that “There is not much you can do to lower your chances of getting cancer,” compared with 16% of those with higher educational attainment (unadjusted *P* = 0.03, adjusted *P* = 0.05); no significant difference was seen between rural and urban participants (Fig. 3; Supplementary Table S4). Of those with lower (vs. higher) educational attainment, 75% versus 60% agreed that there are so many recommendations about preventing cancer, it is hard to know which ones to follow (unadjusted *P* = 0.06, adjusted *P* = 0.04). When asked whether they would rather not know their chances of getting cancer, almost twice as many rural participants agreed (32% vs. 17% urban, unadjusted *P* < 0.0001, adjusted *P* = 0.08). Eighty percent of participants agreed with the statement “There are things I could change in my life to reduce my risk of cancer,” with little variation by rurality or educational attainment; 59% of participants agreed that “It seems like everything causes cancer,” again with little variation by rurality or educational attainment.

**FIGURE 4:**
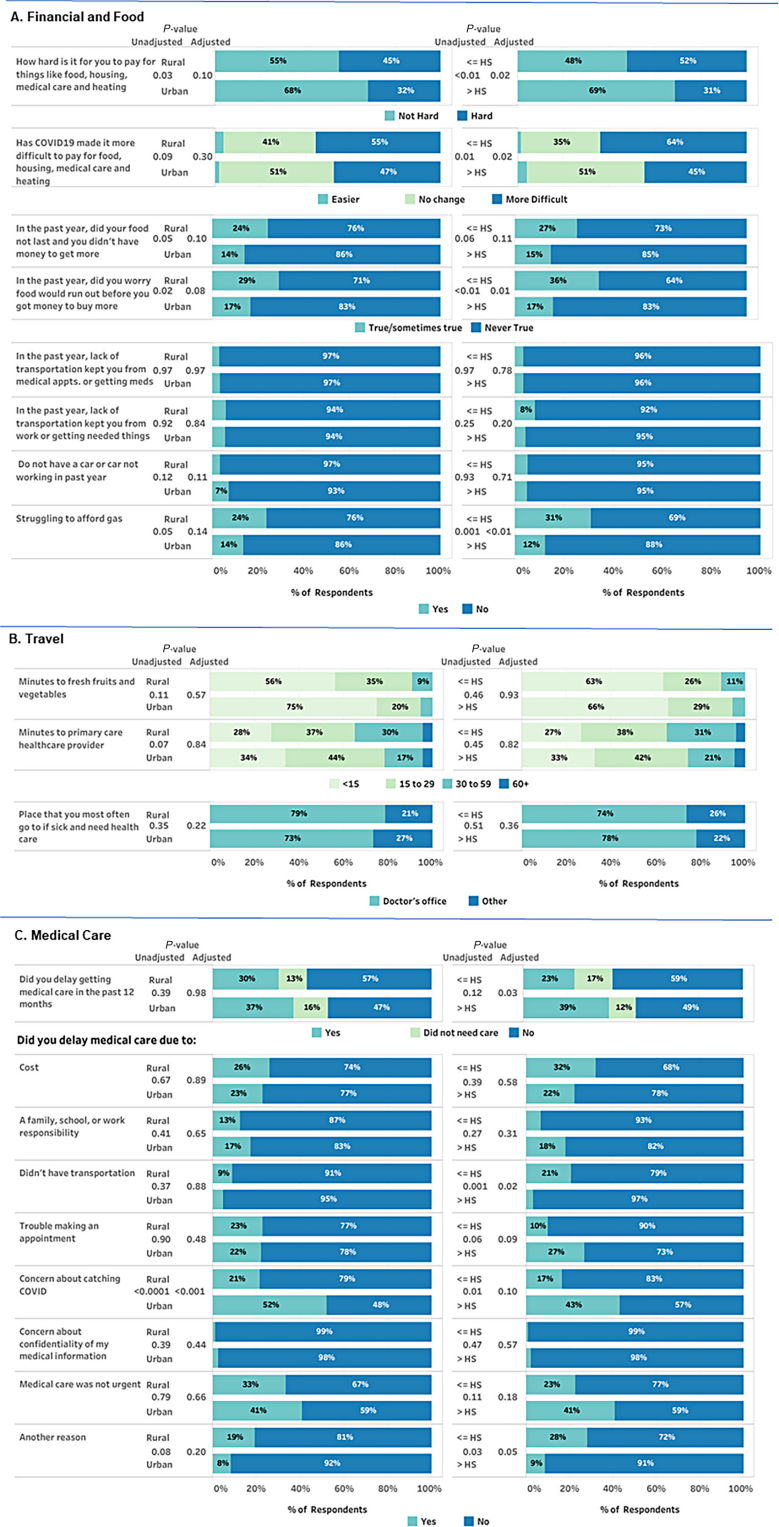
Social determinants of health and influence of COVID-19 pandemic by rurality (rural vs. urban) and educational attainment [≤ high school (HS) vs. > HS] among 1,717 NH and VT residents surveyed in February–March 2022. **A,** Financial and food. **B,** Travel. **C,** Medical care.

Among larger households of three or more people, those living in rural areas were more likely to have a lower income (Table 1). Significantly more participants with higher (79%) than lower (61%) educational attainment were homeowners. Greater proportions of participants with lower educational attainment found it hard to pay for basic necessities like food, housing, medical care, and heating (52% vs. 31%, unadjusted *P* < 0.01, adjusted *P* = 0.02; Fig. 4; Supplementary Table S5). When asked whether the COVID-19 pandemic impacted their ability to pay for these same basic necessities, 64% of those with lower versus 45% of those with higher educational attainment agreed (unadjusted *P* = 0.01, adjusted *P* = 0.02). Rural residence was not significantly associated with financial hardship after adjustment for educational attainment. In univariate analyses, rural populations and those with lower educational attainment were significantly more likely to experience food insecurity, but in the multivariable model, educational attainment was independently associated with concern that the food would run out before getting money to buy more (adjusted *P* = 0.01). Difficulty paying for gas was more often experienced by those with lower (31%) than higher (12%) educational attainment (unadjusted *P* = 0.001, adjusted *P* < 0.01).

One-third of participants reported delaying medical care during the preceding 12 months; these proportions were lower among those with lower educational attainment (23% vs. 39%, adjusted *P* = 0.03; Fig. 4; Supplementary Table S5). Although most participants in the sample reported that reliable transportation was not a barrier to keeping medical appointments, among those who reported missing or delaying medical care within the past year, those with lower educational attainment more often cited transportation as an important cause (21%) compared with those with higher educational attainment (3%, adjusted *P* = 0.02). Interestingly, significantly more urban than rural participants missed or delayed care because of concerns about catching COVID-19 (52% vs. 21%, adjusted *P* < 0.001), but significant differences were not seen by educational attainment after adjustment for rurality (unadjusted *P* = 0.01, adjusted *P* = 0.10). Cost and availability of appointments as reasons for delayed care did not differ significantly by rurality or educational attainment.

## Discussion

In this study, we assessed the independent contributions of educational attainment and rural residence to a variety of risk factors that might influence cancer incidence and mortality. Although univariate analyses identified several cancer risk factors associated with both lower educational attainment and rural residence, we found—as others have done (33–34)—that those with lower educational attainment more often live in rural areas. Our multivariable models indicate that lower educational attainment in NH and VT was the more important independent explanatory factor, and that rural residence did not tend to present significant additional risk either alone or as an interaction term with educational attainment.

In our study, smoking prevalence was more than twice as high in those with high school education or less, and was not independently associated with rural residence. Previous studies have shown that some of the overlap in cancer risk between rural individuals and those with lower educational attainment is likely attributable to smoking (8, 35). We also found that those with lower educational attainment experienced greater financial hardship, and that the COVID-19 pandemic exacerbated these difficulties independent of rural residence. Economic hardship is known to be an important contribution to cancer risk, impacting people's access to healthy foods, preventive health care services, and screening (36–38).

Beliefs about cancer and cancer prevention influence engagement in preventive behaviors (13). We found strong evidence for differences in cancer beliefs by educational attainment. Proportionally, almost twice as many individuals with lower than higher educational attainment agreed that “There is not much you can do to lower your chances of getting cancer”. This question from the Health Information National Trends survey has been described as an attempt to quantify fatalism (12, 16, 39, 40). This presents an important potential target for clearer educational messaging especially in view of our finding that those with lower educational attainment are also more often affected by difficulty knowing which of the many recommendations about cancer prevention they should follow. Our findings support prior work showing higher levels of cancer fatalism and risk information avoidance among those with lower versus higher educational attainment (13, 38). Both cancer fatalism and risk information avoidance have previously been shown to be associated with lower levels of screening and worse outcomes, such as more advanced stage at diagnosis and higher mortality (15, 22, 40–42).

Our survey was conducted at the start of the third year of the COVID-19 pandemic. Anxiety about catching COVID-19 was a deterrent to seeking medical care, and—unlike most of the associations that we found—this concern was independently associated with urban residence but not with educational attainment. Although urban populations—which include a greater proportion of individuals with higher educational attainment—were more open to cancer prevention in principle, their concerns about exposure to COVID-19 in health care environments presented substantial barriers to seeking care, a phenomenon also reported elsewhere (43–44). Now that safe and effective vaccines are widely available, these perceived COVID-19 risk barriers are likely to subside (45). However, others report that COVID-19 appears to have exacerbated other existing barriers to preventive care that may prove more intractable and deepen cancer disparities for years to come (46). Although less anxious about catching COVID-19, those with lower educational attainment in our study experienced other barriers to medical and preventive care—such as fatalistic beliefs, financial hardship, and lack of transportation. The increased financial hardship due to the pandemic reported by this group may have lowered cancer prevention on their list of priorities. Others have also reported that reduced availability of medical care during the pandemic delayed diagnoses and disproportionately affected rural participants and other disadvantaged population subgroups (46–50). It will be important to assess the long-term effects of the pandemic on disparities in cancer risk and outcomes by rurality and education.

Previous studies have highlighted travel time to preventive services as a barrier for rural residents (8, 19–20, 51), and cancer beliefs and inadequate communication about screening as barriers for those with lower educational attainment (12, 14). Both rurality and educational attainment have been associated with insurance and cost barriers (52–54). In our survey, although rural participants had more difficulty paying for gas, food, medical care, and heating than their urban counterparts, the associations with rurality tended to diminish after adjustment for educational attainment. While the financial hardships experienced by those with lower educational attainment place this group at an important disadvantage, we found it surprising that interaction terms in the models did not indicate additional excess risk in this subgroup when also living in rural areas where high-quality medical services are less accessible (28) but this could have been due to insufficient power. In previous studies in NH, travel barriers were significantly associated with choice of treatment for early-stage breast cancer, likely because radiotherapy involves multiple trips to a distant location in a short period of time (55), but uptake of mammography was not significantly related to travel distances (56), likely because breast cancer screening usually involves only a single trip for which the travel distances in NH are not prohibitive.

Several aspects of the study limit its generalizability. The response rate of 22% is low, perhaps in part due to attrition within the survey panels, but higher than response rates typically seen in random digit dialing surveys (57). The populations of NH and VT differ substantially in rurality (40% vs. 71%, respectively). NH's nonrural population is largely confined to small cities in the southeast corner, in only 10% of its land area and its municipalities have a range of economic and social characteristics (58). VT is the most rural state in the nation, with most living rurally and no cities of more than 50,000 people (59). The inclusion of both states may broaden generalizability of our findings to states of varying rurality, although rural areas are known to be heterogeneous in terms of cancer risk behaviors and barriers to care. Urban populations in NH and VT are unlikely to be representative of urban populations in larger cities (12, 17, 52). The states are also racially and ethnically homogenous, with minorities comprising fewer than 10% of their population (60). However, NH and VT are of interest because they have high cancer incidence (NH) and mortality (VT), and particularly high incidence of several cancer subtypes such as bladder and uterine cancers, and melanoma (25–27).

In our region, meaningful disparities remain for those with lower educational attainment who comprise a greater proportion of rural than urban populations. Understanding the independent role of educational attainment in NH and VT will help in the design and implementation of interventions to reduce cancer morbidity and mortality in our most disadvantaged populations. Educational attainment is a determinant of economic status and health (61) and this overlap is important not only because of its causal role but also because of the potential impact on interventional strategies. For example, it is more difficult to reduce smoking prevalence in individuals with lower educational attainment and financial hardship (60–63). Improving access to high-quality education will require fundamental investments in infrastructure including student support services and financial aid (64). While cancer centers and public health partners can play a role in influencing legislative and local policies relating to educational infrastructure, they also have opportunities to address more immediate cancer control targets. For example, The DCC Community Outreach and Engagement team has provided educational messaging at the levels of providers, patients, and community to increase provision and uptake of lung cancer screening; we are codesigning educational tools to increase acceptance of home radon testing; we distribute incentives such as gas cards for disadvantaged individuals; and we now plan to increase our recruitment of individuals with lower educational attainment to project design teams and advisory committees. The important factors that emerged in these analyses—financial hardships, beliefs about cancer prevention, and smoking—represent key potential targets for approaches to remedy disparities in the catchment population, starting by including individuals from disadvantaged subgroups as partners in the investigative teams and design processes.

## Supplementary Material

Supplementary Data T1 Survey instrument

Supplementary Data T2 Comparison of key variables between responders and non responders

Supplementary Data T3 Cancer risk and prevention behaviors by rurality and educational attainment

Supplementary Data T4 Cancer beliefs by rurality and educational attainment

Supplementary Data T5 Social factors by rurality and educational attainment
