# Supplementary material for: A Survey of Cancer Risk Behaviors, Beliefs, and Social Drivers of Health in New Hampshire and Vermont
Source: Cancer Res Commun. 2023 Aug 29;3(8):1678–87. doi: 10.1158/2767-9764.CRC-23-0267 (PMC10464638; doi:10.1158/2767-9764.CRC-23-0267)
Supplement: Supplementary Data T1 — Survey instrument [file crc-23-0267-s01.pdf]

Supplemental Table 1: Survey instrument and sources of any questions used or adapted from external sources

Survey Item	Answer Choices	External sources, where applicable)
What is the zip code of your primary residence?	• N/A	University of New Hampshire Survey Center
And what is your current age?<br>(Please enter a number only)	• N/A	University of New Hampshire Survey Center
How many of the persons who currently live in your household are under 18 years of age, including babies and small children?	• None<br>• One<br>• Two<br>• Three<br>• Four<br>• Five<br>• Six<br>• Seven or more<br>• Don't know/Not sure	University of New Hampshire Survey Center
Including yourself, how many adults currently live in your household?	• None<br>• One<br>• Two<br>• Three<br>• Four<br>• Five<br>• Six<br>• Seven or more<br>• Don't know/Not sure	University of New Hampshire Survey Center
Which of the following kinds of health insurance are you currently covered by?<br><br>*Please select all that apply.*	• Insurance purchased through an employer or union (including plans purchased through another person's employer)<br>• Insurance that you or another family member buys on your own, including through the Exchange (also known as Obamacare or the ACA)<br>• Medicare<br>• Medicaid or other state program<br>• TRICARE (formerly CHAMPUS), VA, or Military<br>• Alaska Native, Indian Health Service, Tribal Health Services<br>• Some other source (Please specify)<br>• I do not have health insurance	Centers for Disease Control and Prevention (CDC). *Behavioral Risk Factor Surveillance System Survey Questionnaire*. Atlanta, Georgia: U.S. Department of Health and Human Services, Centers for Disease Control and Prevention, 2016.

How hard is it for you to pay for things like food, housing, medical care, and heating?	• Very hard<br>• Somewhat hard<br>• Not very hard<br>• Not hard at all	Centers for Medicare & Medicaid Services. (2019). The accountable health communities health-related social needs screening tool. *AHC Screening Tool*.<br><br>Hall, M. H., Matthews, K. A., Kravitz, H. M., Gold, E. B., Buysse, D. J., Bromberger, J. T., . . . Sowers, M. (2009). Race and Financial Strain are Independent Correlates of Sleep in Midlife Women: The SWAN Sleep Study. Sleep, 32(1), 73-82. doi:10.5665/sleep/32.1.73
Has the COVID-19 pandemic made it more difficult or easier to pay for things like food, medical care, and heating?	• Much more difficult<br>• Somewhat more difficult<br>• No change<br>• Somewhat easier<br>• Much easier<br>• Don't know/Not sure	
Please indicate how often this was true for you within the past 12 months:<br><br>The food you bought just **didn't last** and you didn't have money to get more.	• Often true<br>• Sometimes true<br>• Never true<br>• Don't know	Centers for Medicare & Medicaid Services. (2019). The accountable health communities health-related social needs screening tool. *AHC Screening Tool*.<br><br>Hager, E. R., Quigg, A. M., Black, M. M., Coleman, S. M., Heeren, T., Rose-Jacobs, R., Cook, J. T., Ettinger de Cuba, S. E., Casey, P. H., Chilton, M., Cutts, D. B., Meyers A. F., Frank, D. A. (2010). Development and Validity of a 2-Item Screen to Identify Families at Risk for Food Insecurity. Pediatrics, 126(1), 26-32. doi:10.1542/peds.2009-3146.
Please indicate how often this was true for you within the past 12 months:<br><br>You were **worried** that your food would run out before you got money to buy more.	• Often true<br>• Sometimes true<br>• Never true<br>• Don't know	Centers for Medicare & Medicaid Services. (2019). The accountable health communities health-related social needs screening tool. *AHC Screening Tool*.<br><br>Hager, E. R., Quigg, A. M., Black, M. M., Coleman, S. M., Heeren, T., Rose-Jacobs, R., Cook, J. T., Ettinger de Cuba, S. E., Casey, P. H., Chilton, M., Cutts, D. B., Meyers A. F., Frank, D. A. (2010). Development and Validity of a 2-Item Screen to Identify Families at Risk for Food Insecurity. Pediatrics, 126(1), 26-32. doi:10.1542/peds.2009-3146.
What is your living situation today?	• I have a steady place to live<br>• I have a steady place to live today, but I am worried about losing it in the future<br>• I do not have a steady place to live (I am temporarily staying with others, in a hotel, in a shelter, living outside on the street, on a beach, in a car, abandoned building, bus or train station, or in a park)	Centers for Medicare & Medicaid Services. (2019). The accountable health communities health-related social needs screening tool. *AHC Screening Tool*.<br><br>National Association of Community Health Centers and Partners, National Association of Community Health Centers, Association of Asian Pacific Community Health Organizations, Association OPC, Institute for Alternative Futures. (2017). PRAPARE. http://www.nachc.org/research-and-data/prapare/
Which of the following best describes who owns your current home?	• The home I live in is owned by me or my family<br>• I live in a home owned by someone else and pay rent<br>• I live in a home owned by someone else, but I do not pay rent<br>• Other	

In the **past 12 months**, has a lack of reliable transportation kept you from...<br><br>a) going to medical appointments or getting medications<br>b) going to work or getting things needed for daily living	• Yes<br>• No<br>• Don't know/Don't remember	Centers for Medicare & Medicaid Services. (2019). The accountable health communities health-related social needs screening tool. *AHC Screening Tool*. Q15.<br><br>National Association of Community Health Centers and Partners, National Association of Community Health Centers, Association of Asian Pacific Community Health Organizations, Association OPC, Institute for Alternative Futures. (2017). PRAPARE. http://www.nachc.org/research-and-data/prapare/
In the **past 12 months**, which of the following transportation problems have you faced regularly (once a month or more)?	• NO TRANSPORTATION PROBLEMS<br>• Do not have a car or my car is not working<br>• Struggling to afford gas<br>• Do not have any public transportation/ride services in my area<br>• My car is unreliable<br>• Rides with friends are unreliable<br>• Public transportation / Ride share services / Transportation services are unreliable<br>• Cannot drive<br>• Other (Please specify)	
Below, please enter the number of minutes it usually takes you to get to the following services or activities (one way).<br>*If you have never done these things, please estimate how long you believe it would take.*<br><br>a) To get fresh fruits and vegetables for your household<br>b) To go to your healthcare provider for primary care (e.g., check-up or minor medical problem)	• Minutes to travel one-way _____<br>• Does not apply<br>• Don't know/Not sure	
Which of the following is the place that you MOST OFTEN go to if you are sick and need health care?	• Doctors office or health center<br>• Urgent care center<br>• Clinic in a drug store or grocery store<br>• Hospital emergency room<br>• VA medical center or VA outpatient clinic<br>• Some other place ___________<br>• No usual place	National Center for Health Statistics. National Health Interview Survey, 2019. Public-use data file and documentation. https://www.cdc.gov/nchs/nhis/data-questionnaires-documentation.htm. 2020.
Did you delay getting medical care in the **past 12 months**?	• Yes<br>• No<br>• Did not need medical care in the past 12 months<br>• Don't remember/Not sure	
Why did you delay getting medical care in the **past 12 months**?<br><br>*Please select all that apply.*	• Cost<br>• A family, school, or work responsibility<br>• Didn't have transportation<br>• Had trouble making an appointment<br>• Concern about catching COVID-19<br>• Concern about confidentiality of my medical information<br>• Decided to wait because I felt the medical care was not urgent<br>• Other (Please specify)	

In which of the following ways have you communicated with a healthcare provider in the **past 12 months**?<br><br>*Please select all that apply.*	• An audio call on a landline<br>• An audio call on a cellphone<br>• A video call on a cellphone<br>• A text on a cellphone<br>• A video call on a computer<br>• A video call on a television<br>• Email<br>• An online patient portal<br>• In-person office visit<br>• Other (Please specify)<br>• Did not communicate with a healthcare provider in the past 12 months	
How often do you need to have someone help you read or understand instructions, pamphlets, or other written material from your doctor or pharmacy?	• Always<br>• Often<br>• Sometimes<br>• Rarely<br>• Never<br>• Don't know/Not sure	Morris, N.S., MacLean, C.D., Chew, L.D. *et al.* The Single Item Literacy Screener: Evaluation of a brief instrument to identify limited reading ability. *BMC Fam Pract* **7,** 21 (2006). https://doi.org/10.1186/1471-2296-7-21
Since the COVID-19 pandemic began, have you experienced any new symptoms you worried could be cancer?	• Yes<br>• No<br>• Don’t know/Not sure	
And in which of the following ways, if any, has the COVID-19 pandemic affected your ability to get medical help to address these symptoms?<br>*Please select all that apply.*	• I postponed getting medical help for these symptoms<br>• I decided not to get medical help for these symptoms<br>• I decided not to get medical help for these symptoms because they went away<br>• I had difficulty making an appointment<br>• My healthcare provider postponed my appointment to address these symptoms<br>• My healthcare provider canceled my appointment to address these symptoms<br>• I had a telehealth or virtual appointment instead of an in-person appointment to talk about these symptoms<br>• Other (Please specify)<br>• COVID-19 did not affect my ability to get medical help for these symptoms	
In the **past 12 months**, have you delayed getting any of the following?<br><br>a) A breast cancer screening (mammography)<br>b) A colorectal cancer screening (colonoscopy, sigmoidoscopy, or stool testing)<br>c) Cervical cancer screening (pap smear)<br>d) Lung cancer screening (CT scan)	• Yes<br>• No<br>• I was not due for this screening in the past 12 months<br>• Not applicable to me	

Why did you delay getting a breast cancer screening (mammography) in the **past 12 months**?<br><br>*Please select all that apply.*	• Cost<br>• A family, school, or work responsibility<br>• Didn't have transportation<br>• Had trouble making an appointment<br>• Concern about catching COVID-19<br>• Concern about confidentiality of my medical information<br>• Decided to wait because I felt the medical care was not urgent<br>• Another reason (Please specify)	
Why did you delay getting colorectal cancer screening (colonoscopy, sigmoidoscopy, or stool testing) in the **past 12 months**?	• Cost<br>• A family, school, or work responsibility<br>• Didn't have transportation<br>• Had trouble making an appointment<br>• Concern about catching COVID-19<br>• Concern about confidentiality of my medical information<br>• Decided to wait because I felt the medical care was not urgent<br>• Another reason (Please specify)	
Why did you delay getting cervical cancer screening (pap smear) in the **past 12 months**?	• Cost<br>• A family, school, or work responsibility<br>• Didn't have transportation<br>• Had trouble making an appointment<br>• Concern about catching COVID-19<br>• Concern about confidentiality of my medical information<br>• Decided to wait because I felt the medical care was not urgent<br>• Another reason (Please specify)	
Why did you delay getting lung cancer screening (CT scan) in the **past 12 months**?	• Cost<br>• A family, school, or work responsibility<br>• Didn't have transportation<br>• Had trouble making an appointment<br>• Concern about catching COVID-19<br>• Concern about confidentiality of my medical information<br>• Decided to wait because I felt the medical care was not urgent<br>• Another reason (Please specify)	
Have you ever been diagnosed with cancer?	• Yes, and still in active treatment<br>• Yes, but no longer receiving treatment for cancer<br>• No<br>• Prefer not to answer	
Which of the following kinds of cancer have you had or do you currently have?<br><br>*Please select all that apply.*	• Breast cancer<br>• Colorectal cancer<br>• Lung cancer<br>• Skin cancer (melanoma)<br>• Non-melanoma skin cancer (basal cell carcinoma or squamous cell carcinoma)<br>• Prostate cancer<br>• Other (Please specify)	

In which of the following ways, if any, did the COVID-19 pandemic affect your ability to get **treatment** or **follow-up** care for cancer?<br><br>*Cancer treatment refers to surgery, radiotherapy, chemotherapy, or immunotherapy*<br>*Cancer follow-up care refers to scans, appointments, or bloodwork for cancer surveillance after your treatment ends*<br><br>*Please select all that apply*	• I postponed getting one or more of my cancer treatments or follow-up care<br>• I decided not to get one or more of my cancer treatments or follow-up care<br>• My healthcare provider postponed one or more of my cancer treatments or follow-up care<br>• My healthcare provider altered one or more of my cancer treatments or follow-up care<br>• Some of my cancer appointments were telehealth or virtual appointments instead of in-person appointments<br>• Other (Please specify)<br>• COVID-19 didn't affect my cancer treatment or follow-up care<br>• I was not scheduled to receive cancer treatment or follow-up care during the pandemic	
How important would it be to you that a healthcare provider discusses the following cancer **screening** topics with you?<br><br>a) Breast cancer screening<br>b) Cervical cancer screening<br>c) Colorectal cancer screening<br>d) Lung cancer screening	• Very important<br>• Somewhat important<br>• Not very important<br>• Not important at all<br>• Not applicable to me	
How important would it be to you that a healthcare provider discusses the following cancer **prevention** topics with you?<br><br>a) Sun safety<br>b) Tobacco use<br>c) Weight loss<br>d) Healthy eating<br>e) Physical activity<br>f) Alcohol use<br>g) Well-water testing for arsenic or radon<br>h) Home air radon testing<br>i) HPV vaccination<br>j) Other (please specify)	• Very important<br>• Somewhat important<br>• Not very important<br>• Not important at all<br>• Not applicable to me	
Overall, how confident are you that you could get advice or information about cancer if you needed it?	• Very confident<br>• Somewhat confident<br>• Not very confident<br>• Not confident at all<br>• Don't know/Not sure	National Cancer Institute (NCI). *Health Information National Trends Survey Instrument (HINTS 1).* Bethesda, Maryland: U.S. Department of Health and Human Services, National Institutes of Health, National Cancer Institute, 2003.

Now we would like to ask you some of your opinions about cancer. Please indicate whether you agree or disagree with each of the following statements.<br><br>a) It seems like everything causes cancer<br>b) There's not much you can do to lower your chances of getting cancer<br>c) There are so many different recommendations about preventing cancer, it's hard to know which ones to follow<br>d) I'd rather not know my chance of getting cancer<br>e) There are things I could change in my life to reduce my risk of cancer	• Strongly agree<br>• Somewhat agree<br>• Neutral<br>• Somewhat disagree<br>• Strongly disagree<br>• No opinion	National Cancer Institute (NCI). *Health Information National Trends Survey Instrument (HINTS 1).* Bethesda, Maryland: U.S. Department of Health and Human Services, National Institutes of Health, National Cancer Institute, 2003.<br><br>National Cancer Institute (NCI). *Health Information National Trends Survey Instrument (HINTS 2).* Bethesda, Maryland: U.S. Department of Health and Human Services, National Institutes of Health, National Cancer Institute, 2005.<br><br>National Cancer Institute (NCI). *Health Information National Trends Survey Instrument (HINTS 4, Cycle 2).* Bethesda, Maryland: U.S. Department of Health and Human Services, National Institutes of Health, National Cancer Institute, 2012.
Have you smoked cigarettes in the **past 15 years**?	• Yes<br>• No	American Lung Association. *Saved by the Scan Quiz*. Available at https://www.lung.org/lung-health-diseases/lung-disease-lookup/lung-cancer/saved-by-the-scan/quiz?gclid=EAIaIQobChMI0InAltDy_QIVGsmUCR2NagfbEAAYASABEgLrGfD_BwE
How many years have you smoked cigarettes in your life?<br><br>*Please enter whole numbers only.*		
Do you currently smoke cigarettes?	• Yes<br>• No	
About how many cigarettes do you usually smoke each day on average?<br>*There are 20 cigarettes in a pack.*<br><br>*Please enter whole numbers only.*		University of Michigan. *Should I Screen.* Available at: https://shouldiscreen.com/English/pack-year-calculator
When you were smoking cigarettes, about how many cigarettes did you usually smoke each day on average?<br>*There are about 20 cigarettes in a pack.*<br><br>*Please enter whole numbers only.*		University of Michigan. *Should I Screen.* Available at: https://shouldiscreen.com/English/pack-year-calculator
Have you heard of a test to find lung cancer before the cancer creates noticeable problems?	• Yes, I have heard of it and have had it done<br>• Yes, I have heard of it but haven't had it<br>• No, I haven't heard of it<br>• Don't know/Not sure	National Cancer Institute (NCI). *Health Information National Trends Survey Instrument (HINTS 3).* Bethesda, Maryland: U.S. Department of Health and Human Services, National Institutes of Health, National Cancer Institute, 2008.
The next question is about CT or CAT scans. During this test, you lie flat on your back on a table. While you hold your breath, the table moves through a donut-shaped x-ray machine which the scan is done. In the past 12 months, have you had a CT or CAT scan?	• Yes<br>• No<br>• Don't know/Not sure	Centers for Disease Control and Prevention (CDC). *Behavioral Risk Factor Surveillance System Survey Questionnaire*. Atlanta, Georgia: U.S. Department of Health and Human Services, Centers for Disease Control and Prevention, 2019.

And was this CT or CAT scan to check for lung cancer or for another reason?	• To check for lung cancer<br>• For another reason<br>• Don't know/Can't remember	Centers for Disease Control and Prevention (CDC). *Behavioral Risk Factor Surveillance System Survey Questionnaire*. Atlanta, Georgia: U.S. Department of Health and Human Services, Centers for Disease Control and Prevention, 2019.
In an average week, how many days do you engage in moderate or more intensive exercise (like walking fast, jogging, running, swimming, or biking) for at least 30 minutes?	• 0<br>• 1<br>• 2<br>• 3<br>• 4<br>• 5<br>• 6<br>• 7	Centers for Medicare & Medicaid Services. (2019). The accountable health communities health-related social needs screening tool. *AHC Screening Tool*.<br><br>Coleman, K. J., Ngor, E., Reynolds, K., Quinn, V. P., Koebnick, C., Young, D. R.,…Sallis, R. E. (2012). Initial Validation of an Exercise "Vital Sign" in Electronic Medical Records. Medicine and Science in Sport and Exercise, 44(11), 2071-2076. doi:10.1249/MSS.0b013e3182630ec1
In **an average week**, how many **days** do you have at least one drink of any alcoholic beverage such as beer, wine, a malt beverage or liquor?	• 0<br>• 1<br>• 2<br>• 3<br>• 4<br>• 5<br>• 6<br>• 7	Centers for Disease Control and Prevention (CDC). *Behavioral Risk Factor Surveillance System Survey Questionnaire*. Atlanta, Georgia: U.S. Department of Health and Human Services, Centers for Disease Control and Prevention, 2019.
And about how many drinks would you say you have **in total** in a given week?<br><br>*Please enter whole numbers only.*		
When you are outside for more than one hour on a warm, sunny day, how frequently do you do the following things?<br><br>a) Wear long pants<br>b) Wear a hat that shades your face, ears, and neck<br>c) Wear a shirt with sleeves that cover your shoulders<br>d) Stay in the shade or under an umbrella<br>e) Wear sunscreen	• Always<br>• Often<br>• Sometimes<br>• Rarely<br>• Never<br>• Don't know/Not sure	National Cancer Institute (NCI). *Health Information National Trends Survey Instrument (HINTS 2).* Bethesda, Maryland: U.S. Department of Health and Human Services, National Institutes of Health, National Cancer Institute, 2005.
Where does the drinking water for your home come from?	• Town/city water<br>• Private well<br>• Other (Please specify)<br>• Don't know/Not sure	
Has your home's well water been tested for arsenic by a lab?	• Yes<br>• No<br>• Don't know/Don't remember	Flanagan SV, Marvinney RG, Zheng Y. Influences on domestic well water testing behavior in a Central Maine area with frequent groundwater arsenic occurrence. *Sci Total Environ*. 2015;505:1274-1281. doi:10.1016/j.scitotenv.2014.05.017
Was the arsenic level measured for your well within normal range or was it high?	• It was normal<br>• It was high<br>• Don't know/Don't remember	

Which of the following actions did you take because of the arsenic level in your well water?<br><br>*Please select all that apply.*	• Installed a filter on tap or under sink<br>• Installed a filter for the whole house<br>• Used more bottled water<br>• Used more water from a jug filler, or a filter in the fridge<br>• Something else (Please specify)<br>• None	Flanagan SV, Marvinney RG, Johnston RA, Yang Q, Zheng Y. Dissemination of well water arsenic results to homeowners in Central Maine: influences on mitigation behavior and continued risks for exposure. *Sci Total Environ*. 2015;505:1282-1290. doi:10.1016/j.scitotenv.2014.03.079
Why did you not take any arsenic-related action?<br><br>*Please select all that apply.*	• Too expensive<br>• Not concerned about arsenic level<br>• Didn't know what to do<br>• Didn't know who to contact<br>• It is not my home<br>• Other reason (Please specify)	Flanagan SV, Marvinney RG, Johnston RA, Yang Q, Zheng Y. Dissemination of well water arsenic results to homeowners in Central Maine: influences on mitigation behavior and continued risks for exposure. *Sci Total Environ*. 2015;505:1282-1290. doi:10.1016/j.scitotenv.2014.03.079
Has the air in your home been tested for radon?	• Yes<br>• No<br>• Don't know/Don't remember	
Would you like to receive a free test that you can use to test your home for radon?<br><br>*(If you answer yes, we will send you an email with the information on how to receive the free test)*	• Yes<br>• No	
Was the radon test result within normal range or high?	• Normal *(less than 4 pCi/L)*<br>• High *(greater than 4 pCi/L)*<br>• Don't know/Don't remember	
Was the radon test result low normal or high normal?	• Low Normal *(less than 2 pCi/L)* – No action needed<br>• High Normal *(between 2- 4 pCi/L)* – with a recommendation to consider fixing the home<br>• Don't know/Don't remember	
Was the radon test result high or very high?	• High *(between 4 - 10pCi/L)*<br>• Very High *(greater than 10 pCi/L)*<br>• Don't know/Don't remember	
Since you got your radon test result, which of the following has happened regarding a radon mitigation system (usually a type of fan that's installed under your home in the foundations to move air to the outside of the home)*?*	• A new radon mitigation system has been installed in my home<br>• An existing mitigation system has been improved in my home<br>• No changes have been made to my home's existing radon mitigation system<br>• There is still no radon mitigation system in my home<br>• Other (Please specify)<br>• Don't know/Not sure	Aloise-Young, Patricia & Cross, Jennifer & Sieving, Gwen. (2015). Increasing Residential Radon Mitigation Rates: A CBSM Study (Final Report). 10.13140/RG.2.2.25110.75843.
Why did you decide not to take radon-related action?<br><br>*Please select all that apply.*	• Too expensive<br>• Not concerned about radon level<br>• Didn't know what to do<br>• Didn't know who to contact<br>• It is not my home<br>• Other reason (Please specify)	Flanagan SV, Marvinney RG, Johnston RA, Yang Q, Zheng Y. Dissemination of well water arsenic results to homeowners in Central Maine: influences on mitigation behavior and continued risks for exposure. *Sci Total Environ*. 2015;505:1282-1290. doi:10.1016/j.scitotenv.2014.03.079
On average, how often do you use a wood stove or wood-burning fireplace **within a living area** of your home?	• 0 (I do not use a wood stove or fireplace in a living area within my home)<br>• 1 to 2 days per week during cold months<br>• 3 to 4 days per week during cold months<br>• 5 to 7 days per week during cold months<br>• Other (specify)	

On average, how often do you use a pellet stove **<u>within a living area</u>** of your home?	• 0 (I do not use a wood stove or fireplace in a living area within my home)<br>• 1 to 2 days per week during cold months<br>• 3 to 4 days per week during cold months<br>• 5 to 7 days per week during cold months<br>• Other (specify)	
Are you currently married, widowed, divorced, separated, or have you never been married?	• Married<br>• Widowed<br>• Divorced<br>• Separated<br>• Never married<br>• Living together	University of New Hampshire Survey Center
Which of the following best describes your gender?	• Woman<br>• Man<br>• Transgender<br>• Gender expansive<br>• Prefer not to say	University of New Hampshire Survey Center
Which of the following ethnic or racial groups do you identify with? (Please select all that apply)	• Native American, Inuit, or Aleut<br>• Asian American/Pacific Islander<br>• African American/Black/Caribbean American<br>• Caucasian/White<br>• Latin/Hispanic<br>• Other – Specify<br>• Prefer not to say	University of New Hampshire Survey Center
What is the highest grade in school or level of education that you've completed and got credit for?	• Eighth grade or less<br>• Some high school<br>• High school graduate (includes G.E.D.)<br>• Technical school<br>• Some college<br>• College graduate<br>• Postgraduate work<br>• Don't know/Not sure	University of New Hampshire Survey Center
Are you registered to vote at your current address?	• Yes<br>• No<br>• Don’t know/Not sure	University of New Hampshire Survey Center
And what are you registered as?	• Registered Democrat<br>• Registered Independent/Unaffiliated/Undeclared<br>• Registered Republican<br>• Registered Other<br>• Don't know/Not sure	University of New Hampshire Survey Center
Generally speaking, do you usually think of yourself as a Republican, a Democrat, an Independent, or something else?	• Democrat<br>• Independent<br>• Republican<br>• Other party<br>• Don't know/not sure	University of New Hampshire Survey Center
Which party do you think of yourself as closer to?	• Republican Party<br>• Democratic Party<br>• Neither<br>• Don't know/Not sure	University of New Hampshire Survey Center

In the election for President in **2020** did you vote for Donald Trump, Joe Biden, Jo Jorgensen, someone else, or did you skip that election?	• Donald Trump<br>• Joe Biden<br>• Jo Jorgensen<br>• Other<br>• Did not vote<br>• Don't know/Not sure	University of New Hampshire Survey Center
How much **total** income did you and your family receive in 2021, not just from wages or salaries but from **all** sources, that is, before taxes and other deductions were made?	• Less than $15,000 (Less than $1,250 per month)<br>• $15,000-$29,999 ($1,250-$2,499 per month)<br>• $30,000-$44,999 ($2,500-$3,749 per month)<br>• $45,000-$59,999 ($3,750-$4,999 per month)<br>• $60,000-$74,999 ($5,000-$6,249 per month)<br>• $75,000-$99,999 ($6,250-$8,333 per month)<br>• $100,000-$149,999 ($8,334-$12,499 per month)<br>• $150,000-$199,999 ($12,500-$16,666 per month)<br>• $200,000 and over ($16,667 and over per month)<br>• Don't know/Not sure	University of New Hampshire Survey Center

Where no external source is cited, questions were developed by the study team
