# Supplementary material for: A Survey of Cancer Risk Behaviors, Beliefs, and Social Drivers of Health in New Hampshire and Vermont
Source: Cancer Res Commun. 2023 Aug 29;3(8):1678–87. doi: 10.1158/2767-9764.CRC-23-0267 (PMC10464638; doi:10.1158/2767-9764.CRC-23-0267)
Supplement: Supplementary Data T2 — Comparison of key variables between responders and non responders [file crc-23-0267-s02.docx]

Supplemental Table 2. Comparison of key variables between responders and non responders

| Variable | Responders | Non responders | Overall Chi^2^ p value |
| --- | --- | --- | --- |
| Gender  Female  Male  Other  Missing/unknown | 45.9%  48.0%  1.7%  4.3% | 43.8%  38.4%  1.3%  16.5% | <0.001 |
| Political party affiliation  Democrat  Independent  Republican  Missing/unknown | 56.7%  11.4%  23.4%  8.5% | 39.5%  9.9%  19.3%  31.1% | <0.001 |
| Educational attainment  High school or less  Technical school/some college  College graduate  Postgraduate degree  Missing/unknown | 4.6%  14.8%  30.0%  44.4%  6.2% | 6.2%  17.3%  23.6%  23.0%  29.9% | <0.001 |
| Age  18-34  35-49  50-64  65+  Missing/unknown | 5.5%  13.5%  31.3%  39.4%  10.3% | 6.0%  11.6%  15.7%  14.2%  52.6% | <0.001 |

Note that the weighting process in the analysis addresses imbalances between responders and the distribution of these factors in the general population
